# Risk of Herpes Simplex virus type 1 (HSV-1) among patients with insomnia: A retrospective cohort study

**DOI:** 10.1097/MD.0000000000039112

**Published:** 2024-08-02

**Authors:** Chun-Ping Lin, Chia-Ling Lin, Hsueh-Ching Wu, Chi-Hsiang Chung, Wu-Chien Chien

**Affiliations:** a Department of Nursing, Shu-Zen Junior College of Medicine, and Management, Kaohsiung, Taiwan; b Department of Nursing, Chang Gung University of Science and Technology, Taoyuan, Taiwan; c Department of Nursing, Hsin Sheng Junior College of Medical Care and Management, Taoyuan, Taiwan; d School of Public Health, National Defense Medical Center, Taipei, Taiwan; e Taiwanese Injury Prevention and Safety Promotion Association (TIPSPA), Taipei, Taiwan; f Department of Medical Research, Tri-Service General Hospital and National Defense Medical Center, Taipei, Taiwan; g Graduate Institute of Science, National Defense Medical Center, Taipei, Taiwan.

**Keywords:** Cohort study, Herpes simplex virus type 1, infection, Insomnia, National Health Insurance Research Database

## Abstract

The aim of this study was to explore the risk of Simplex virus type 1 (HSV-1) in patients with insomnia. This study applied a population-based retrospective cohort design. A total of 50,210 patients aged ≥ 20 years who had received a diagnosis of insomnia between 2000 and 2015. They were identified according to the corresponding International Classification of Diseases, Ninth Revision, Clinical Modification (ICD-9-CM) code. The control cohort comprised 100,420 age-matched and sex-matched patients. Data from the Taiwan National Health Insurance Research Database were employed from 2000 to 2015. The overall incidence of HSV-1 in the insomnia cohort was significantly higher than that in the comparison cohort (3.10 vs 0.33 per 1000 person-years). Patients with insomnia had a higher risk of HSV-1 infection, compared with the comparisons (hazard ratio (HR) = 4.33, 95% confidence interval (CI) 2.18–5.58). For individuals divided into 3 age groups (≤40, 41–65, and >65 years old), the HSV-1 infection risk of the insomnia cohort was significantly greater than that of the comparisons. As the duration of insomnia increases, the risk of HSV-1 occurrence decreases.

**Key points**

1. The overall incidence of HSV-1 in the insomnia cohort was significantly higher than that in the control cohort (3.10 vs 0.33 per 1000 person-years).
2. Patients with insomnia had a higher risk of HSV-1 infection than the controls.
3. As the duration of insomnia increases, the risk of HSV-1 occurrence decreases.

## 1. Introduction

An epidemiological study estimated that over 3.7 billion people (67%) under the age of 50 have been infected with HSV-1.^[1]^ The prevalence of HSV-1 in adults in the United States and Taiwan is 47.8% and 63.2%, respectively.^[2,3]^ HSV-1 infections are common and lifelong, with clinical symptoms that include painful blisters or ulcers at the site of infection,^[4]^ and they affect the quality of life.^[5]^ The triggers of HSV-1 are not completely understood, but age, socioeconomic, stress, ethnicity and immune dysfunction have been supported as risk factors.^[5,6]^ HSV-1 is an annoying health problem that is often ignored.^[7]^

Chronic insomnia has demonstrated a significant association with the risk of infectious diseases,^[8,9]^ inference linked to decreased immunity function,^[10]^ or influence the potential control of Interleukin-6 responses,^[11]^ elevate inflammatory markers^[12]^ and increasing the probability of infection disease.^[13]^

There are very few investigations on the correlation between insomnia and HSV-1. Only Meshreky et al^[14]^ found no significant difference in the frequency of HSV-1 infection in older adults with and without insomnia, indicating no correlation between sleep disorders and HSV-1 infection. In Asian cultures, it is believed that oral herpes simplex is attributed to “heatiness” or an excess of “liver fire,” and it is suggested to be more prevalent in cases of poor sleep quality.^[15,16]^ Previous study was limited to the older adults, study design and did not explore the correlation between the duration of insomnia and HSV-1, more research evidence is needed to support the association between sleep problems and HSV-1. Hence, this study investigates the risk of HSV-1 in patients with insomnia compared to those without insomnia, utilizing a large dataset from Taiwan’s National Health Insurance Database (NHIRD).

## 2. Methods

### 2.1. Data source

The data source for our study is the Longitudinal Health Insurance Database 2005 (LHID2005), a dataset from Taiwan’s National Health Insurance Research Database (NHIRD). LHID2005 data were collected through systematic and random sampling of NHIRD data; the database includes data on 2 million people. There was no significant difference in the gender and age distribution between the patients in the randomly sampled data and the original NHIRD.^[17]^

In the LHID2005 dossier, disease diagnosis codes are based on the International Classification of Diseases, Ninth Revision, Clinical Modification (ICD-9-CM).^[18]^ Due to the patient identifiers were encrypted before their data were applied for study purposes to protect confidentiality, the requirement for written or verbal consent from patients for data linkage was waived. This database has demonstrated consistent reliability in numerous nationwide cohort studies.^[19,20]^ The Ethics Review Board of Tri-Service General Hospital (TSGHIRB No E202316043) approved this study.

### 2.2. Study design and participants

This study employed a retrospective cohort study design. The study cohort consisted of patients diagnosed with insomnia between 2000 and 2015. They were identified according to the corresponding ICD-9 code (780.52; insomnia, unspecified). The inclusion criteria for the study cohort were: age ≥ 18 years; outpatient and emergency room diagnosis codes for insomnia 3 or more times, or hospitalization diagnosis codes at least once; use of hypnotics, with diagnoses confirmed by psychiatrists.

**Figure 1.**
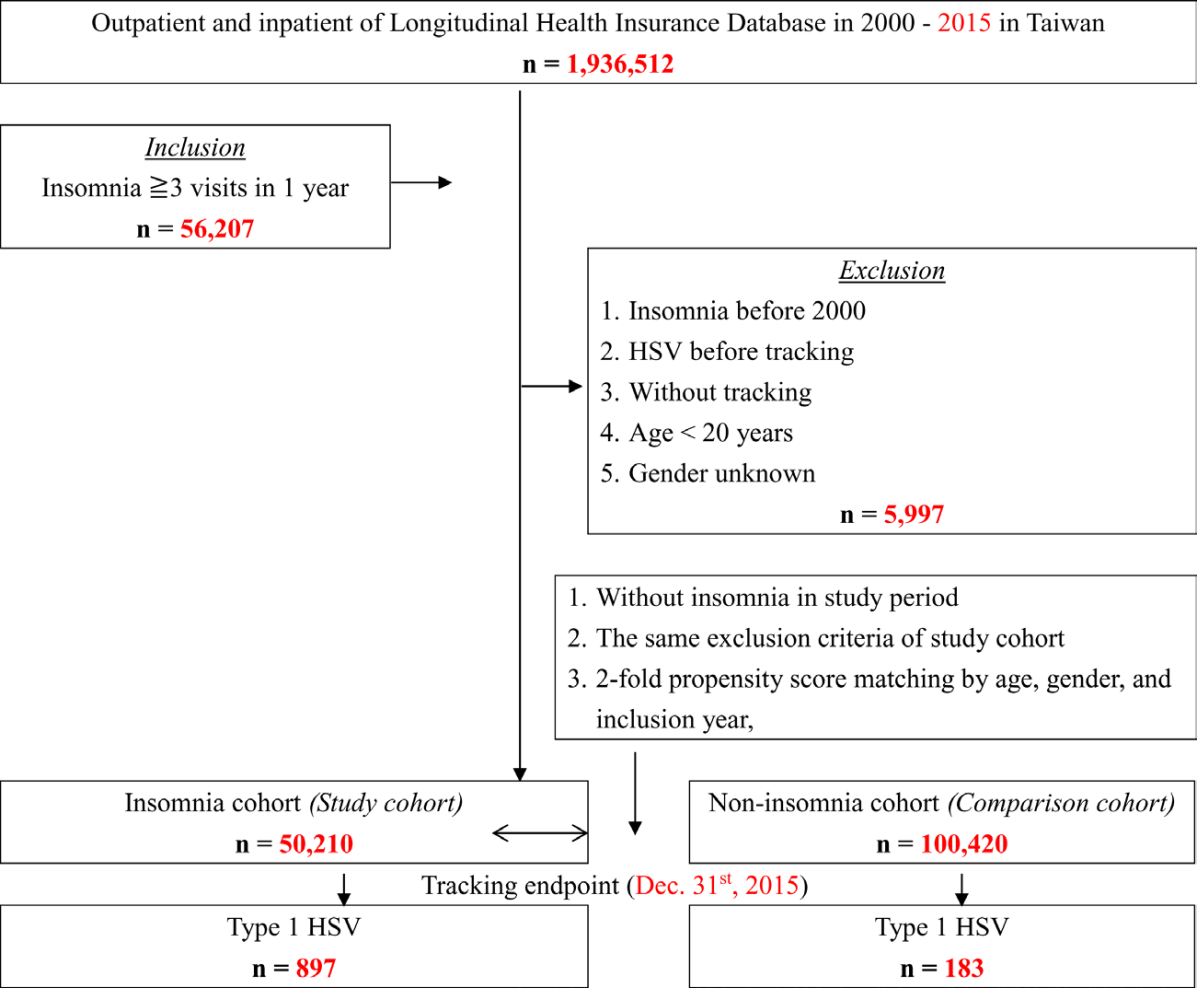
The flowchart of study sample selection from National Health Insurance Research Database in Taiwan.

From subjects without insomnia in the Longitudinal Health Insurance Database 2005, a comparison cohort was assembled by matching each insomniac with 2 non-insomnia individuals on index enrollment date, sex, and age. To avoid potential confounding effects, we excluded those with previous diagnoses of insomnia or HSV-1 before the index enrollment date in both the study cohort and the comparison cohort (Fig. 1).

### 2.3. Outcome measures

All study individuals were followed up from the index date until either the onset of HSV-1 (ICD-9 054.9) in the subset file from the LHID2005,^[21]^ withdrawal from the NHI program, or the end of 2015 (whichever occurred first).

### 2.4. Definitions of other variables

The individuals were grouped by sex, urbanization level (low, middle, and high), according to their address registered with the National Health Insurance (NHI), and season of diagnosis outcome (spring, March–May; summer, June–August; autumn, September–November; and winter, December–February).

The comorbidities considered in this study were hyperlipidemia (ICD-9-CM codes 272), type 2 diabetes (ICD-9-CM codes 250), hypertension (ICD-9-CM codes 401–405), stroke (ICD-9-CM codes 430–438), CAD (ICD-9-CM codes 410–414), obesity (ICD-9-CM codes 278.0), anxiety (ICD-9-CM codes 300.0, 300.2, 300.3, 300.83, and 309.81), and depression (ICD-9-CM codes 296.2, 296.3, 300.4, and 311). Patients with any of the conditions were included in the study.

### 2.5. Statistical analysis

All analyses were performed using SPSS version 21 (SPSS, Inc., Chicago, IL). Descriptive statistics were used for basic information, including the percentage, mean, and standard deviation. Chi-squared and *t* tests were used to evaluate the distributions of categorical and continuous variables between the insomnia and control cohorts. The incidence densities of HSV-1 were calculated according to age, sex, urbanization level, season of diagnosis outcome, and comorbidity. Cox proportional hazards regression models were used to determine the risk of HSV-1, and the results are presented as hazard ratios (HRs) with 95% confidence intervals (CIs). The same variables were used in a multivariable analysis. The HSV-1risk difference between the 2 groups was estimated using the Kaplan–Meier method with the log-rank test. A 2-tailed *P* value of <.05 indicated statistical significance.

## 3. Results

### 3.1. Demographic data

In Table 1, the insomnia and control cohorts comprised 50,210 and 100,420 patients, respectively. In study cohort, most patients were less 65 years (45.97%). The main comorbidity in both cohorts was HT. Most patients were classified as living in an area with a middle level of urbanization. The average follow-up duration was 8.22 (SD 6.67) years.

**Table 1:** Comparison of demographics between insomnia patients and non-insomnia cohort in the endpoint.

Variables	Insomnia cohort (n = 50,210)		Non-insomnia cohort (n = 100,420)		*P* value
	n	%	n	%	
Age (yr)					
≤40	7772	15.48	18,883	18.80	<.001***
41 to 65	19,356	38.55	33,995	33.85	
>65	23,082	45.97	47,542	47.34	
Age (Mean ± SD)	62.17 ± 17.89		62.81 ± 18.55		<.001***
Gender					
Male	22,179	44.17	44,358	44.17	.999
Female	28,031	55.83	56,062	55.83	
Low-income					
No	48,931	97.45	97,979	97.57	.170
Yes	1279	2.55	2441	2.43	
Season					
Spring (March–May)	11,675	23.25	27,259	27.14	<.001***
Summer (June–August)	13,630	27.15	26,163	26.05	
Autumn (September–November)	16,182	32.23	28,198	28.08	
Winter (December–February)	8723	17.37	18,800	18.72	
Urbanization level					
Highest	11,724	23.35	34,481	34.34	<.001***
Middle	22,584	44.98	47,540	47.34	
Lowest	15,902	31.67	18,399	18.32	
Comorbidities					
Hyperlipidemia	1265	2.52	2412	2.40	.167
Diabetes	8244	16.42	16,367	16.30	.551
Hypertension	9557	19.03	18,655	18.58	.032*
Stroke	4491	8.94	8942	8.90	.798
CAD	5038	10.03	9730	9.69	.034*
Obesity	39	0.08	44	0.04	.010*
Anxiety	1134	2.26	350	0.35	<.001***
Depression	2820	5.62	808	0.80	<.001***
Follow-up period (yr)	8.19 ± 6.64		8.24 ± 6.71		.171

The average follow-up period of study was 8.22 ± 6.67 (yr).

* *P *< .05.

** *P *< .01.

*** *P *< .001.

### 3.2. Incidence rates and adjusted HR of HSV-1 by age, sex, urbanization level, and comorbidities

Table 2 reveals that during the follow-up period, the risk of HSV-1 was 4.33 times higher in the insomnia cohort than in the controls (adjusted HR, 95% CI, 2.18–5.58).

**Table 2:** Comparison of IR and adjusted HR of Type 1 HSV between with insomnia and non-insomnia cohort by demographic characteristics and comorbidity.

Variables	Insomnia cohort			Non-insomnia cohort			Crude HR (95% CI)	Adjusted HR† (95% CI)
	Events	PYs	IR#	Events	PYs	IR#		
Overall	897	289,485	3.10	183	546,560	0.33	5.31 (3.12–6.31)***	4.33 (2.18–5.58)***
Age (yr)								
≤40	128	35,315	3.62	13	56,243	0.23	8.74 (7.71–9.31)***	7.06 (6.18–8.63)***
41 to 65	224	106,555	2.10	59	163,597	0.36	4.32 (2.99–5.84)***	3.22 (1.68–4.77)***
>65	545	147,615	3.69	111	326,720	0.34	5.33 (3.18–6.35)***	4.39 (2.26–5.63)***
Gender								
Male	480	123,373	3.89	128	225,881	0.57	4.47 (2.96–5.94)***	3.89 (1.96–5.04)***
Female	417	166,112	2.51	55	320,679	0.17	5.67 (3.89–6.72)***	5.01 (2.97–6.74)***
Low-income								
No	851	273,108	3.12	176	534,219	0.33	5.35 (3.22–6.44)***	4.38 (2.25–5.70)***
Yes	46	16,377	2.81	7	12,341	0.57	5.12 (3.06–6.17)***	4.08 (2.18–5.33)***
Season								
Spring	272	56,423	4.82	46	126,880	0.36	6.94 (4.82–8.06)***	5.12 (2.35–6.02)***
Summer	157	71,135	2.21	62	132,687	0.47	3.22 (1.88–4.25)***	3.01 (1.54–4.17)***
Autumn	209	96,230	2.17	40	158,880	0.25	4.68 (2.97–5.82)***	4.26 (2.01–4.96)***
Winter	259	65,697	3.94	35	128,113	0.27	5.46 (3.19–6.38)***	4.38 (2.22–5.61)***
Urbanization level								
Highest	202	58,746	3.44	69	157,469	0.44	5.37 (3.18–6.37)***	4.38 (2.25–5.64)***
Middle	367	121,166	3.03	74	246,720	0.30	4.82 (2.97–6.24)***	4.28 (2.06–5.31)***
Lowest	328	109,573	2.99	40	142,371	0.28	5.96 (3.30–6.48)***	4.71 (2.56–5.88)***
Comorbidities‡								
No	600	154,432	3.89	113	290,771	0.39	4.86 (2.72–5.80)***	3.35 (1.62–4.24)***
Yes	297	135,053	2.20	70	255,789	0.27	5.86 (3.92–6.84)***	5.00 (3.24–5.97)***

Crude HR, hazard ratio.

#IR, incidence rate, per 1000 person-years;

† Adjusted HR: multivariable analysis including age, gender, low-income, season, urbanization level, and comorbidities.

‡ Comorbidity: Only to have one of comorbidities (including hyperlipidemia, diabetes, hypertension, stroke, CAD, obesity, anxiety, depression) classified as the comorbidity group.

*P < .05.

**P < .01.

*** *P *< .001.

When the patients were divided into 3 age groups (≤40, 41–65, and >65 years), the risk of HSV-1 in the insomnia cohort was significantly higher for all ages groups. For sex, both men and women in the insomnia cohort were at a significantly higher risk of HSV-1, compared with the controls. Other variables including low-income, season, urbanization level, comorbidities association with the risk of HSV-1.

### 3.3. Comparisons of incidence and risk of HSV-1 by insomnia duration

**Figure 2.**
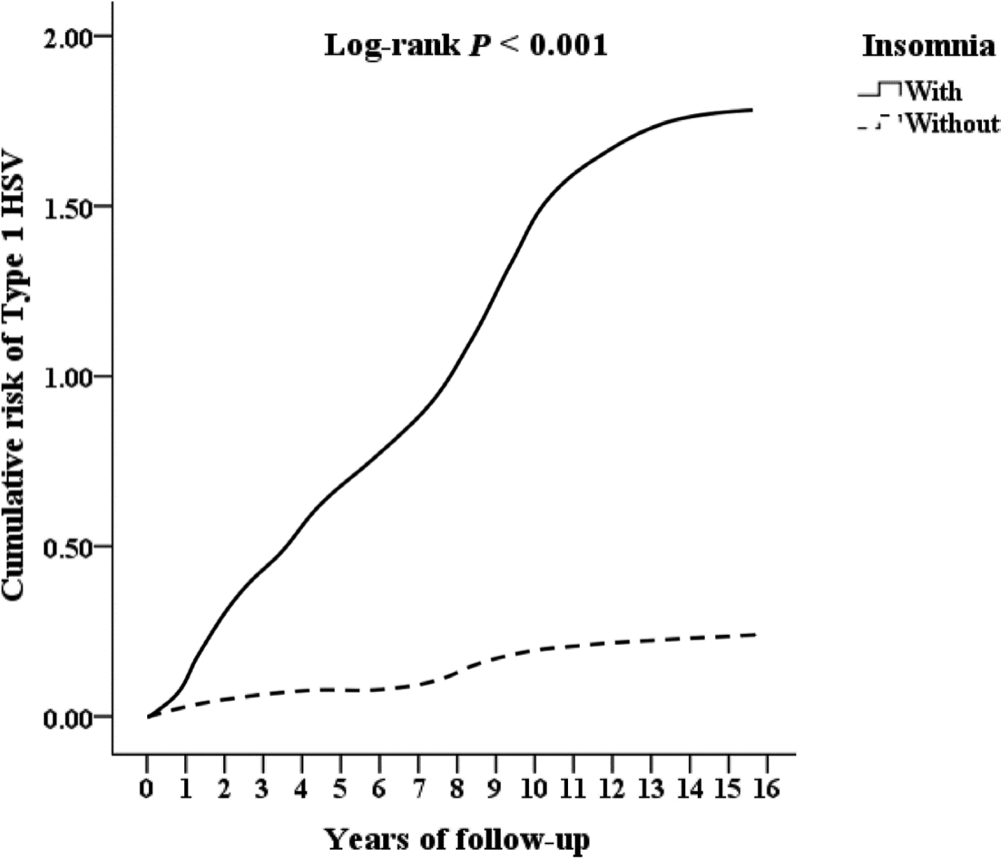
Kaplan–Meier for cumulative risk of Type 1 HSV among aged 20 and over stratified by insomnia with log-rank test.

We conducted a Kaplan–Meier survival analysis to assess the cumulative incidence of HSV-1 and discovered significant differences between the 2 cohorts (log-rank test; *P* < .001) (Fig. 2).

Table 3 presents comparisons of incidence and risk of HSV-1 stratified by insomnia duration. In the study cohort, patients with durations of both <4 years, 4 to 6 years and >6 years were at a significantly higher risk of HSV-1, compared with the controls, particularly <4 years of duration with insomnia group, the higher the risk of the occurrence of HSV-1.

**Table 3:** Comparisons of incidence and risk of Type 1 HSV by insomnia duration.

	N	Event	IR#	Crude HR (95% CI)	Adjusted HR† (95% CI)
Non-insomnia cohort	100,420	183	0.33	1.00 (Reference)	1.00 (Reference)
Insomnia duration					
<4 yrs	18,432	572	5.74	8.13 (6.25–9.78)***	7.83 (5.20–8.93)***
4 to 6 yrs	8973	140	3.13	5.33 (3.17–6.39)***	4.35 (2.24–5.67)***
>6 yrs	22,805	185	1.61	3.26 (1.54–4.47)**	3.01 (1.10–4.26)*

Crude HR, hazard ratio.

#IR, incidence rate, per 1000 person-years.

† Adjusted HR: multivariable analysis including age, gender, low-income, season, urbanization level, and comorbidities.

* *P *< .05.

** *P *< .01.

*** *P *< .001.

The definition of insomnia duration: diagnostic (ICD-9-CM 780.53) ≥ 3 visits in 1 yr.

## 4. Discussion

Present study found insomnia patients had 4.33 times (*P* < .001) the risk of HSV-1 than comparisons. In addition to, this study provides the effect tended to decrease with the insomnia duration of follow-up, when exposure insomnia duration less 4 years, 4 to 6 years, and more 6 years, the risk of HSV-1 was 7.83 (*P* < .001), 4.35 (*P* < .001), and 3.01 (*P* < .05) times, compare with comparisons. These results indicated the risk of HSV-1 associated with the insomnia by retrospective cohort study. According to our literature, this is the first longitudinal population-based study on the risk of HSV-1 associated with insomnia in patients in Asian population.

Although the clear mechanism underlying the association remains unclear, inferences to results can be made from other studies to explain the interpretation. Prior studies supported that sleep problems have significant influence on the risk of infectious disease,^[11]^ which may lead to affect immune system makers, such as interleukin-6, tumor necrosis factor-α,^[22]^ or increased the circulating levels of C-reactive protein, may be induced that inflammatory processes are activated.^[23]^ In addition, circadian rhythms disturbances associated with HSV-1 risk, sleep loss closed relationship with chronic fatigue syndrome, is associated with HSV-1 prevalence.^[24,25]^ These biological theories could support the current findings that insomnia had a higher risk of developing HSV-1.

The present results found that subjects over 65 years with insomnia had the highest risk of HSV-1 (3.69 cases per 1000 person-years). A previous European study indicated that HSV-1 prevalence increased with age, reflecting cumulative exposure.^[26]^ It’s worth noting that this study’s results showed that subjects aged ≤ 40 years had an HSV-1 risk of 3.62 cases per 1000 person-years. The prevalence of HSV-1 infection increases gradually from childhood, reaching 70% to 80% in adulthood.^[27]^ Another study indicated 47.8% of persons aged 14 to 49 was infected with HSV-1.^[2]^ Symptoms of HSV-1 infection generally include gingivostomatitis and vesicular or ulcerative lesions on the oropharyngeal mucosa, HSV-1 can affect hundreds of millions of people, decreasing the quality of life.^[28]^ Therefore, we suggest focusing on young adults’ sleep behaviors and the susceptibility to HSV-1 in further study.

This study revealed the association between insomnia duration and HSV-1 risk, was particularly pronounced in those with insomnia duration < 4 years’ group. We inference to subjects with insomnia, may long-term use of hypnotics, the severity of insomnia may generally relief, decreasing the adverse effects of insomnia on HSV-1 risk. In addition, a study found that subjects with continual insomnia exposure were associated with adaptive changes in immunity.^[22]^ A previous study found circadian rhythmic fluctuations over the course of a short period that impact the susceptibility to or progression of the viral infection.^[29]^ However, in our study, we could not confirm the possible mechanism because of the tolerance effects in the immune response. We suggest that further studies focus on the potential adverse physiological effects of insomnia on HSV-1.

### 4.1. Study limitations

This study has several limitations. First, the NHIRD could not provide detailed material on lifestyle and sleep behaviors, such as physical activities, sleep duration or sleep quality. These factors could be potentially associated with the results, but we were unable to obtain these data for our analyses because of no information in the NHIRD. Second, patients with insomnia may have been underrepresented because patients with insomnia might not accept medical treatment and could not record in databank. Third, a previous study indicated that the overall concordance between claims records and self-reported survey results was moderate for clinical diagnoses,^[30]^ we cannot exclude the possibility of unmeasured or unknown confounding factors. These factors may introduce potential biases, which could compromise the results. Therefore, we suggest exploring the relationship between insomnia and HSV-1 using a prospective cohort study design in future research. Forth, we might have underestimated the incidence of HSV-1, as we known the antiviral medications, such as acyclovir,^[31]^ are the most effective medications available for people infected with HSV-1, if subjects’ self-pay medications from pharmacy, their data were not recorded in the NHIRD, the prevalence of HSV-1 could be underestimate.

From a public health prevention perspective, the findings of this study can provide clinical care professionals with an understanding of the close association between chronic insomnia and the increased risk of HSV-1. It is crucial to emphasize to high-risk groups with sleep problems the important role that sleep plays in preventing HSV-1 infection. By enhancing patient education on sleep hygiene and providing strategies to improve sleep quality, the incidence of HSV-1 could be effectively reduced. These results can significantly contribute to disease prevention and health promotion efforts.

## 5. Conclusion

In summary, this is the study to explore the association between insomnia and HSV-1. The present study provides valuable information that insomnia is associated with an increased risk of HSV-1, which is essential for health care practitioners to be informed of. The approach of the HSV-1 prevention needs to improve the sleep quality.

## Author contributions

**Conceptualization:** Chun-Ping Lin, Chia-Ling Lin, Hsueh-Ching Wu, Wu-Chien Chien.

**Data curation:** Chun-Ping Lin, Chia-Ling Lin, Hsueh-Ching Wu, Wu-Chien Chien.

**Formal analysis:** Chun-Ping Lin, Chia-Ling Lin, Hsueh-Ching Wu, Chi-Hsiang Chung, Wu-Chien Chien.

**Supervision:** Chun-Ping Lin.

**Writing – original draft:** Chun-Ping Lin, Chia-Ling Lin.

**Methodology:** Chia-Ling Lin.

**Writing – review & editing:** Hsueh-Ching Wu, Wu-Chien Chien.

**Software:** Chi-Hsiang Chung.
